# Review of Rapid Diagnostic Methods for *Vibrio Cholerae* Detection in the Last Decade (2011–2022)

**DOI:** 10.24248/eahrj.v7i2.724

**Published:** 2023-11-30

**Authors:** Mwangi Harrison Ndung'u, George Gachara, Lameck Ontweka, Nelson Menza, Abednego Musyoki, Margaret Muturi

**Affiliations:** a Department of Medical Laboratory sciences, Kenyatta University, Nairobi, Kenya; b Department of Biochemistry, University of Nairobi, Nairobi, Kenya

## Abstract

**Introduction:**

Cholera epidemic poses a global public health threat, heavily impacting the global economy and societies, with Africa and Asia particularly affected due to factors like; inadequate sanitation, contaminated water, and overcrowding. The associated high rates of morbidity and mortality strain productivity and healthcare costs while complicating control measures. Consequently, the World Health Organization's cholera control initiative and the Diarrhoeal Diseases Laboratory Network advocate for rapid responses to outbreaks and continuous environmental surveillance, since traditional cholera detection methods relying on phenotypic fingerprinting, although considered the gold standard, suffer from labour-intensiveness, time-consumption, and skill requirements. This results in inadequate surveillance and delayed treatment in remote areas lacking well-equipped laboratories.

**Methods:**

A systematic review was conducted to evaluate the development and performance of cholera rapid diagnostic techniques for detecting cholera in clinical samples and for environmental surveillance purposes over the past decade.

**Results:**

Twenty-four commercially produced diagnostics were identified in January 2011. Ten more were mentioned in the literature and yet did not provide enough relevant data due to suspected production withdrawal or fall-back. The vast bulk of tests were discovered to be based on antigen or antibody detection, with DNA accounting for a large proportion of the residual tests. This study revealed a plethora of diagnostic methods, some of which have not yet made it to the commercial market. Promising approaches, such as; Loop-mediated isothermal amplification (LAMP), ELISA, and simplified PCR protocols, are likely to play a significant role in future cholera screening. Findings are herein summarised in tables and figures.

**Conclusion:**

Cholera epidemic continues to present a formidable global health challenge with economic and social repercussions. Traditional detection methods fall short in resource-limited areas, necessitating the exploration of advanced molecular techniques, like aptamers, to improve diagnosis, surveillance, and control measures, especially in regions vulnerable to cholera outbreaks.

## INTRODUCTION

Cholera, a long-standing global health issue, continues to inflict substantial morbidity and mortality in Low- and Middle-Income countries (LMICs), predominantly in regions of Africa and Asia. Cholera outbreaks are intrinsically linked to challenges such as overcrowding, inadequate and contaminated water supply, and poor sanitation, thus creating an enduring public health crisis.^14,30^ Despite advancements in healthcare, Africa still bears a disproportionate burden of cholera deaths, reflecting global health inequalities. In Kenya, cholera outbreaks persist annually, as evidenced by the 38 cases reported in Garissa and Turkana Counties as of August 8, 2021, emphasising the persistent challenges in effectively implementing outbreak surveillance.^35^

Efforts to control cholera have grown increasingly viable due to several key factors. Availability of oral cholera vaccines, heightened public awareness of water and sanitation, widespread access to Oral Rehydration Salts (ORS) in remote areas, improved communication networks, and active participation by governments and local communities in cholera-prone regions collectively render the strategy for cholera control more attainable.

Cholera is a highly infectious disease, primarily caused by *Vibrio cholerae* serogroup O1 or O139, which triggers severe symptoms like profuse vomiting and “rice watery” diarrhoea.^12,42^ Left untreated, cholera can lead to rapid and severe dehydration, often resulting in death within a mere 3 to 4 hours of symptom onset.^25^ The bacterium's ability to persist in water supplies, aquatic ecosystems, and inadequate sewage disposal, combined with its short incubation period, significantly contributes to cholera's propensity for rapid and deadly outbreaks.^25^ Recent trends indicate a rising number of cholera incidents in vulnerable regions, amplifying its threat.^12^ The major culprit in this infectious drama is the Cholera Toxin (CT), produced by *V. cholerae*. CT is encoded by the ctxAB operon, which is an integral part of the CTX genetic element.^32,37^

The World Health Organization (WHO) initiated a global strategy in 2017 called the “Global Roadmap to 2030.” The primary aim is to reduce cholera-related deaths by 90% and ultimately eliminate cholera from 20 countries.^1,6,43^ This strategic approach emphasises various measures, including early detection and rapid responses to outbreak situations. It recognises that the success of these strategies in achieving cholera control in industrialised countries, through improvements in sanitation, water systems, and general hygiene, cannot be directly applied to developing nations due to cost constraints. As a result, the focus is on countries coming together to pool resources and tackle cholera through collective efforts.^6,15^

Currently, the gold standard for laboratory diagnosis of cholera relies on culture, however, this is increasingly becoming impractical due to the extended time required (often 3 or more days) on selective growth media. Culture, while necessary for phenotypic analysis, antibiotic susceptibility testing, and detailed molecular characterisation essential for global disease surveillance, struggles with delayed detection.^34^ Delays in identifying cholera outbreaks can lead to unfavourable public health responses, disease spread, and increased morbidity and mortality.^37^ Early and prompt interventions are vital for managing and preventing outbreaks.^38^

The Rapid Diagnostic Test (RDT) offers a promising solution. When combined with the enrichment of Alkaline Peptone Water (APW), RDT demonstrates diagnostic performance comparable to culture. It presents an efficient and sustainable alternative, particularly in settings with limited laboratory capacity.^34^

To streamline molecular methods for cholera detection, it is essential to utilise the widely recognised Polymerase Chain Reaction (PCR).^20^ PCR offers several advantages, such as; simplicity, cost-effectiveness, and precision, making it an ideal tool for swift VTA gene detection. This multiplex PCR method not only identifies the presence of VTA genes but also effectively distinguishes *V. cholerae* bacteria from other Vibrio species and bacteria.^24^ It's capable of detecting 10 to 100 Colony Forming Units (CFU) *V. cholerae* and as little as 8.5 to 85 pg of genomic DNA. For the specific detection of cholera toxin, the ctxA gene encoding, conventional coupled PCR or real-time PCR is a suitable choice, with fluorescence-based automated product detection. This approach targets the preferred subunit of cholera toxin.^20^

Multiplex PCR takes cholera typing a step further by enabling the differentiation of *V. cholerae* O1 biotypes. It achieves this by exploiting the sequence differences between the classic biotype and El Tor biotype tcpA genes.^20,24^ This method is cost-effective, offering an alternative to conventional PCR.^3^ Moreover, a quadruplex PCR system has been developed for the simultaneous detection of genes specific to *Vibrio cholerae* O1 and/or O139, including wbe and/or wbf, the cholera toxin A subunit (ctxA), toxin coregulated pilus (tcpA), and central regulating protein ToxR (toxR) – all in a single-tube reaction.^18^

A thermostabilised triplex PCR test simplifies cholera detection further.^18^ This test is conveniently pre-packaged and does not require cold storage. It efficiently identifies both toxigenic and non-toxigenic strains of *V. cholerae* based on the cholera toxin A (ctxA) and outer-membrane lipoprotein (lolB) genes. Additionally, it incorporates an internal amplification control to check for PCR inhibitors in samples. These simplified molecular techniques represent significant advancements in cholera detection, offering efficient and cost-effective solutions.^8^

A cutting-edge method known as Particle Difusometry-Loop-Mediated Isothermal Amplification (PD-LAMP) offers rapid, sensitive, and robust cholera detection in environmental water samples. This technique, using *V cholerae* as a model due to its naturally low concentration in such samples, demonstrates its capability to detect as few as 1 *V. cholerae* cell in molecular-grade water in just 20 minutes. Remarkably, it achieves this without the need for any prior enrichment or sample preparation steps.^9^ Furthermore, by implementing simplified sample preservation techniques and applying Multi-Locus Variable-Number Tandem-Repeat Analysis (MLVA) for the differentiation of *V cholerae* genotypes, researchers can efficiently gather critical information about the genetic diversity of *V. cholera*. This not only advances cholera detection but also enhances our understanding of the disease's genetic variations.^11^

The objective of this systematic review is to assess the performance of cholera rapid detection techniques used in the last decade. Given the urgent need for effective rapid diagnostic methods for proper management and preventive measures, especially in resource-constrained regions, this study aims to identify and evaluate these techniques. The study also focuses on exploring alternative methods to traditional culture-based diagnosis, which is time-consuming, and aims to provide insights into potentially more efficient and cost-effective molecular methods, such as Polymerase Chain Reaction (PCR). Ultimately, this research seeks to contribute to the early detection and containment of cholera outbreaks and epidemics.

## METHODS

A systematic review was conducted to evaluate the development and performance of cholera rapid diagnostic techniques for detecting cholera in clinical samples and for environmental surveillance purposes over the past decade.

### Search

Eight scientific databases, (PubMed, SCOPUS, EMBASE, LILACS, Science Direct, Google Scholar, Medline Plus, and Research GATE) were used for systematic searches (December 20, 2021). The following search strategy was used: Since 2011, all published English literature focusing on diagnostic tools for human clinical samples of cholera, as well as the evaluation of diagnostic tests, has used the initial terms “Cholera”[Mesh] OR “*Vibrio cholerae*”. [Mesh] OR cholerae OR choleras OR cholera OR “Cholera Toxin” [Mesh] AND “Sensitivity and Specificity” [- Mesh] OR “Diagnosis”[Mesh] OR “Diagnosis” [Subheading] OR (routine AND (test OR tests or testing)) [TIAB] OR (false AND ((positive or positivity) or negative)) [TIAB] OR diagnos* [TIAB] in PubMed.^2,12^ Additionally, we manually searched the articles included in the reference lists of the selected studies, as well as related key reviews and cholera RDT technical guidance.^44^

### Eligibility Criteria

A manual search of journals with multiple publications on the subject, such as the Journal of Clinical Microbiology, Transactions of the Royal Society of Tropical Medicine and Hygiene, Biosensors and Bioelectronics, and Journal of Microbiological Methods was conducted. To gather information about commercial diagnostic tools, we conducted a search in grey literature sources, including manufacturer and governmental regulatory websites. We specifically focused on diagnostic tools designed for identifying *Vibrio cholerae* in clinical specimens or environmental samples for surveillance.

### Study Limitation

It is important to note that this study focused exclusively on English-language publications, which might have led to the omission of certain detection techniques.

### Information Source

**FIGURE 1:**
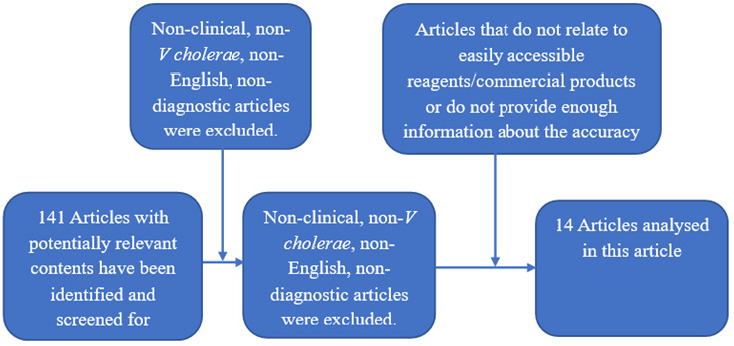
*Vibrio Cholerae* articles selection criteria

This review was limited to publications from 2011 onwards to account for technological advancements following the 2011 cholera outbreaks and the emergence of the new strain O139. Papers were excluded if they did not centre around the identification of *Vibrio cholerae* in clinical or environmental samples. For the eligible studies, we extracted details regarding the identification limit, diagnostic target, techniques used (e.g.; microscopy, agglutination, ELISA, immunochromatography, or PCR), cycle time, intended use, and usage settings during analysis. Laboratory experiments provided information on test sensitivity and specificity, with additional insight from field studies, if available. Field studies with a sample size of over 100 stool samples, reporting Positive Predictive Value (PPV) and Negative Predictive Value (NPV), were reviewed and their results tabulated. The supporting evidence for the validity and reproducibility of RDTs was also evaluated. This comprehensive approach allowed us to assess the landscape of cholera rapid diagnostic techniques in the last decade, shedding light on their development and performance. (Figure 1)

## RESULTS

### Literature Search

#### Diagnostic Tests

Twenty-four commercially produced diagnostics were identified in January 2011. Ten more were mentioned in the literature and yet did not provide enough relevant data due to suspected production withdrawal or fall-back. A clear path of transition is depicted, following closely the historical development of diagnostic innovations in this field from cell culture and microscopy methods to agglutination methods, immunochromatographic assays, and PCR-based assays. The vast bulk of tests were discovered to be based on antigen or antibody detection, with DNA accounting for a large proportion of the residual tests.

Table 1 highlight both the positive and negative predictive values tests for over 100 samples that were evaluated under field conditions, excluding tests where this information was insufficiently reported. *DFA = Direct Fluorescent Antibody, IP = Institute Pasteur, COAT = Coagglutination Test, VC = Vibrio cholerae, SMART=Sensitive Membrane Antigen Rapid Test.*

**TABLE 1:** Positive and Negative Predictive Values Tests for Evaluated Samples

Product name	Positive Predictive Value	Negative Predictive Value
COAT	100	95
IP cholera dipslick	95.6	100
IP cholera dipstick	94.8	98.6
IP cholera dipstick	86	93.3
SMART	84	84
IP dipslick	83	88
Medicos	71	90

DFA = Direct Fluorescent Antibody, IP = Institute Pasteur, COAT = Coagglutination Test, VC = Vibrio cholerae, SMART=Sensitive Membrane Antigen Rapid Test.

**Figure 2:**
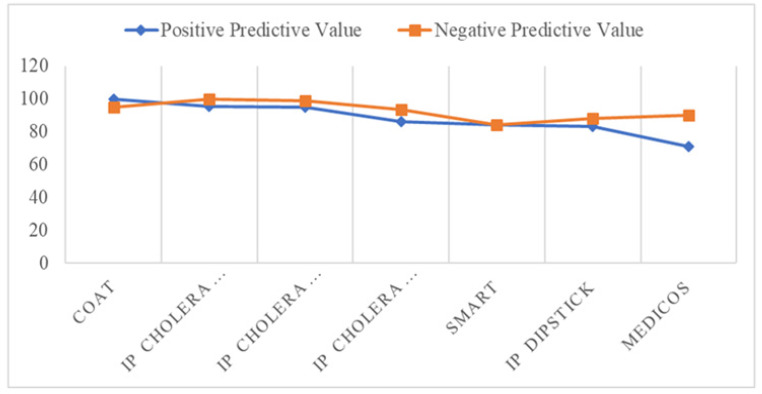
Positive and Negative Predictive Values for Selected Diagnostic Kits Chart

Figure 2 correlations between the negative and positive values of the cholera diagnostic kits. The positive and negative predictive values test for over 100 samples that were evaluated under field conditions, excluding tests where this information was insufficiently reported., IP = Institute Pasteur.

IP = Institute Pasteur

The current review has revealed a plethora of diagnostic methods, some of which have not yet made it to the commercial market. In addition, emerging technologies like microarrays and electro-chemiluminescence are under development as biosensors for detecting cholera toxin and other antigens. Promising approaches, such as; Loop-mediated isothermal amplification (LAMP), ELISA, and simplified PCR protocols, are likely to play a significant role in future cholera screening.^4,7,13,33,39,41^ However, these advanced techniques are beyond the scope of this paper.

To summarise the study's findings, Table 1 and Figure 2 is presented, divided into two sections. The first section covers the positive and negative predictive values for samples. Table 2 examines the specificity and sensitivity values for other molecular diagnostic methods and immunoassays. Each technique is described based on information provided by the manufacturer, which includes; product specifications, intended use, and performance. This data can be invaluable for making comparisons, assessing analytical sensitivity based on detection limits, and evaluating test efficiency based on cycle times. It is important to note that these tests have not been designed or recommended for use with environmental samples; they are primarily intended for prepared samples.

**TABLE 2:** Showing Selected Rapid Diagnostic Kits Sensitivity and Specificity

Product name	Specificity value	Sensitivity Value
Cholera Screen	100	100
Cholera SMART	100	100
Bengal Screen	100	100
Cholera SMART	100	100
DFA COLTA	100	100
COAT	100	100
Cholera SMART	100	98.5
IP cholera dipstick	96	98
SMART	95	97
IP cholera dipstick	92.5	95.6
Bengal DFA	89	95
IP O1 cholera dipstick	89	95
IP cholera dipstick	84	94.2
Medicos	79	92
Cholera Screen	77.8	85.7
Crystal VC	73.5	84
IP dipstick	67	83
Cholera Screen	60	58

### Rapid Test Diagnostic Evaluations

Since 2011, 18 analytical and experimental assessments and correlations of retail diagnostic techniques for the detection of *Vibrio cholerae* in clinical specimens have been conducted. (Figures 1 and Figure 2). These assessments have taken place all over the globe, with a substantial majority taking place in the Indian subcontinent. The largest study enrolled approximately 400 cases and control systems, while the majority of other sample cohorts were rather small, with the lowest containing only 30 patient samples.^12^ The peer-reviewed assessments of the previously stated diagnostic tests performed between 2011 and 2022 are summarised in Tables 2. Table 2 summarises the tests performed in the field.

Fourteen distinct diagnostics were discovered to be analysed and are thus evaluated by comparison in this study. Tables 1, 2 and 3 rated the tests that were assessed in the field with more than 100 samples, with the exception of tests with inadequately observed positively and negatively predicted results, namely; Coagglutination Test (COAT), Institute Pasteur (IP) cholera dipstick, Sensitive Membrane Antigen Rapid Test (SMART), IP dipstick, and Medicos. Tables 2 and 3 present the sensitivities and specificities of the aforementioned 18 evaluations by rank of test in a similar manner.

**TABLE 3:** Pooled Sensitivity and Specificity of Cholera RDTs Using Direct Stool Testing

Test	Data point (n)	Sample size (n)	Pooled sensitivity (95% Cl), %	Pooled specificity (95% Cl), %	Positivity LR (95% Cl)	Negative LR (95% Cl)	DOR (95% Cl)
SD BIOLINE VC 01/0139 Ag RDT.Kit/20	5	1920	90	89	6	0.12	38
Crystal Cholera RDT, dipstick, kit/10	15	9813	91	75	4	0.13	31
Bioline Cholera Ag 01/0139, kit/20	4	1892	89	87	7	0.12	56
All	24	12627	91	80	5	0.11	40

Abbreviations: DOR, diagnostic odds ratio; LR, likelihood ratio; RDT, rapid diagnostic test

## DISCUSSIONS

RDT accuracy using direct specimen testing of cholera was estimated using 18 data points (from over 12,000 specimens). Crystal VC, Cholkit, IP, Artron, and SD Bioline were among the RDTs used. The combined sensitivity and specificity were averaged at 91 and 80%, respectively (Table 2 and 3). The positive LR was 5, the negative LR was 0.11, and the DOR was 40.^2^ A positive LR of 5 indicates that a cholera patient is 5 times more likely than an uninfected individual to have a positive test result. A negative LR of 0.11, on the other hand, indicates that a cholera patient is 9 times (1/0.11) less likely to have a negative test result.

The aggregated sensitivity of 91% indicates that cholera RDTs could very well miss 9% of instances. These failed cases may not profit from cholera calibration and validation and may not be isolated, posing a risk of disease transmission to others. The consolidated sensitivity prediction of cholera RDTs is consistent with the Global Task Force on Cholera Control's (GTFCC) proposed minimum sensitivity performance of 90%.^28^ Nevertheless, the precision (80%) fell short of the GTFCC specificity target of more than 85%.^2,28^ When the proof is examined thoroughly, it appears that these tests are not precise to guide the diagnosis of cholera.

However, because the specificity of different cholera RDTs varies, this should not be extrapolated to all cholera RDT brands. For example, the specificity was greater than 85% in 3 of the analyses (3/11; 27) that used crystal VC. Furthermore, Cholkit RDT's consolidated specificity of 87% which meets the GTFCC specificity target of greater than 85%. To date, only 3 studies^5,19,40^ undertaken in Bangladesh and Malawi have tested the effectiveness of the Cholkit RDT; all of them have been industry-sponsored. This calls for a second Cholkit RDT evaluation to see if the promising optimised precision can be replicated in other studies.

Specificity projections improved substantially in studies that used APW enrichment, but sensitivity forecasts did not. Although this enhancement step takes at least 4 hours, it is still faster than its predecessor technique, which is frequently time-consuming (48 to 72 hours). This description is critical for physicians to remember when cholera RDTs are the only tools available to guide the diagnosis. Given that APW-enriched experiments are not surely instantaneous; it is expected that this inspection (i.e. improved specificity via APW) will be an appealing target for future studies involving APW. The decrease of unfounded positive test results is beneficial for cholera monitoring, i.e. cholera RDTs should be extremely specific to quickly drive triage of samples for stool culture and avoid unnecessary resource mobilisation.^16,17^ Given the ease of use of cholera RDTs in field conditions to quickly determine results, this study fills a knowledge gap in the evidence about the efficacy of cholera RDTs.

We have affirmed that cholera RDTs are vital public health aids that could enable timely detection and surveillance of epidemics and thus allow for a speedy action to control their spread, especially in remote areas and in resource-limited settings. It is possible to use these cholera RDT tools to provide prompt guidance on accurate cholera diagnosis in order to meet the GTFCC's ambitious goal of reducing cholera deaths by 90% by 2030.^22,27^ This analysis has some limitations, one of which is the “imperfect reference standard” used in the majority of the studies. The use of stool culture as a reference standard may have reduced the performance of cholera RDTs. It is feasible that there are stark differences between the outcomes of stool culture and cholera RDTs within certain conditions. However, it is widely mentioned that the cholera RDT outcome may reflect the actual result rather than the stool culture because the bacterial culture is not 100% sensitive. It has the potential to influence the specificity of cholera RDTs due to false-negative culture results; that is, traditional microbiological culture sometimes misclassifies some true cholera cases as not having the disease.^25,27^ Lytic vibriophage, reduced quantity of viable *V. cholerae* in a stool sample due to earlier antibiotic treatment, specimen storage issues, a time lag among sample collection and delayed processing, and the operator's skills,^35^ are all factors that significantly influence stool culture. When the amount of *V. cholerae* in a sample is low, the culture method may fail to detect *V. cholerae*. Although pre-stool antibiotic treatment may interfere with *V. cholerae* detection using RDTs, this assumption is debatable.^10,23,26,31,34^ Antibiotics administered prior to testing may result in a complete absence of live organisms in the stool sample, raising the potential of securing false negative culture results; however, the same stool sample lacking live bacteria may contain high levels of lipopolysaccharide antigens and yield a positive outcome using cholera RDTs.^26,27,31^ Using Crystal VC, Ley et al. discovered no significant difference between patients treated with antibiotics (sensitivity 93.8% and specificity 59.5%; n 14 58) and those who were not (sensitivity 92.8% and specificity 49.3%; n 14 519).^23,28^ Similar studies conducted in Zambia and South Sudan discovered that including or excluding patients who had previously taken antibiotics from the analysis had no effect on the performance of RDTs.^34,29^ There are few studies that have dived into this subject. As a result, it was impossible to resolve this issue.

Besides that, some systematic flaws were discovered in the included studies. Most studies, in particular, did not provide information on patient exclusion. Another limitation is that the sources of suboptimal specificity of cholera RDTs were not investigated in this study. Nevertheless, these limitations would not affect the value of cholera RDTs in monitoring efforts.

## CONCLUSION

Currently, the performance of available RDTs for cholera varies significantly, with most of them underperforming when compared to PCR and culture tests. Some promising non-commercialised RDTs have shown excellent results but require further development to make them available for wider use. RDTs are valuable for swiftly distinguishing cholera from other causes of diarrhoea, especially in remote areas, aiding local containment efforts. However, their role in cholera surveillance is not well-defined. In cases of environmental cholera, other disposable tests like ELISA-based ETEC systems can help pinpoint the cause and prevent unnecessary antibiotic use among the local population in endemic regions.

While PCR is valuable in identifying the cause of diarrheal illnesses and controlling antibiotic resistance, it may not always detect cholera in such samples. Nevertheless, it can contribute to reducing medical costs and enhancing local management.
